# Size of myocardial fibrosis by LGE, pre- and post-contrast T1 and ECV in patients with hypertrophic cardiomyopathy referenced to normal appearing myocardium or healthy volunteers

**DOI:** 10.1186/1532-429X-17-S1-P268

**Published:** 2015-02-03

**Authors:** Maxim Avanesov, Dennis Säring, Ulf K Radunski, Kai Muellerleile, Enver Tahir, Gerhard Adam, Gunnar Lund

**Affiliations:** University Medical Center Hamburg-Eppendorf, Hamburg, Germany

## Background

Myocardial fibrosis is associated with abnormal cardiac remodeling and poor prognosis in patients with hypertrophic cardiomyopathy. Currently, new cardiac MRI (CMR) techniques such as T1-mapping and extracellular volume (ECV) measurement are available to quantify diffuse myocardial fibrosis. We analyzed the size of myocardial fibrosis using pre- and post-contrast T1, ECV and late gadolinium enhancement (LGE) in patients with HCM referenced to normal appearing myocardium and referenced to normal values of healthy volunteers.

## Methods

CMR was performed in 21 patients with HCM (56±4.6 years,10 women) using a 1.5T scanner (Achieva, Philips). Myocardial lesions were assessed on 3 representative short axes of the apex, center and basis of the left ventricle by phase-sensitive inversion-recovery (PSIR) LGE-images, pre- and post-contrast T1 maps and ECV maps. Size of fibrosis was quantified in percent of left ventricular (LV) myocardium by a threshold method relative to normal appearing myocardium using a cutoff >2 SD above normal appearing remote myocardium on all images and relative to normal values assessed from 20 healthy volunteers on T1 and ECV maps. Post-contrast images were obtained after injection of 0.075 mmol/kg Gd-BOPTA. Data were analyzed using the HeAT software.

## Results

**Figure 1:**
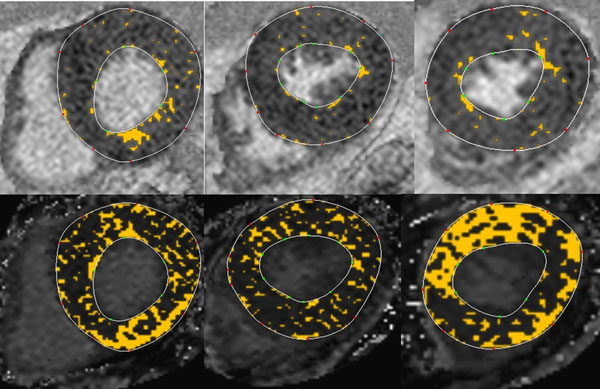
**Upper row:** Basal, central and apical areas of LV on short axes PSIR-LGE images. Lower row: Corresponding ECV maps of the same basal, central and apical areas of LV on short axes. The colored regions of the LV myocardium represent myocardial injury referring to normal myocardium obtained from healthy controls.

Size of fibrosis relative to remote normal appearing myocardium was 20±15%LV on LGE images and 17±16%LV on ECV maps (*p*=0.49). Smaller fibrosis sizes were found on native T1 maps with 14±12%LV (p=0.017) and on post-contrast T1maps with 11±08%LV (*p*=*0.02*). When referenced to normal values of healthy volunteers size of myocardial fibrosis was significantly lager with 35±4%LV on native T1, 49±26%LV on post-contrast T1 and 61±16%LV on ECV maps compared to values referenced to normal appearing myocardium (*P*<0.01, Figure 1). Mean ECV referenced to normal appearing myocardium was with 62±17% larger compared to ECV of 42±12%, when referenced to healthy volunteers (*p*<*0.01*).

## Conclusions

When referenced to normal appearing myocardium similar fibrosis sizes were obtained by LGE and ECV, but smaller fibrosis sizes were measured by native and post-contrast T1. Significantly larger fibrosis sizes were found with all mapping techniques when measurements were referenced to normal values of healthy volunteers, indicating a much larger fibrosis burden in HCM patients than currently observed with LGE imaging.

